# Primary care for the urban poor in India during the pandemic: Uninterrupted management of non-communicable diseases and home-based care of patients with COVID-19 infection

**DOI:** 10.3389/fpubh.2022.1043597

**Published:** 2023-01-09

**Authors:** Sunil Abraham, Sushil Mathew John, Archna Gupta, Seema Biswas, Manorama M. Khare, Pavan Mukherjee, Augustine C. Frankline

**Affiliations:** ^1^Department of Family Medicine and LCECU, Christian Medical College and Hospital, Vellore, India; ^2^Department of Family and Community Medicine, University of Toronto, Toronto, ON, Canada; ^3^BMJ Case Reports, London, United Kingdom; ^4^Department of Family and Community Medicine, University of Illinois College of Medicine Rockford, Rockford, IL, United States

**Keywords:** pandemic (COVID-19), urban poor communities, primary care, India, disaster response

## Abstract

**Problem:**

The two waves of COVID-19 severely affected the healthcare system in India. The government responded to the first wave with a strict nationwide lockdown which disrupted primary care, including the management of non-communicable diseases (NCDs). The second wave overwhelmed healthcare facilities leading to inadequate access to hospital services. Collectively, these issues required urgent responses, including the adaptation of primary care.

**Approach:**

The Low-Cost Effective Care Unit (LCECU) of Christian Medical College, Vellore (CMC) has a network of community volunteers, community health workers, an outreach nurse, social workers and doctors who operate clinics in six poorer areas of Vellore. The network adapted quickly, responding to the lockdown during the first wave and ensuring ongoing primary care for patients with non-communicable diseases. During the second wave, the team developed a system in collaboration with other CMC departments to provide home-based care for patients with COVID-19.

**Local setting:**

The LCECU is a 48-bed unit of the Department of Family Medicine, part of the 3,000-bed CMC. It originated in 1982, aiming to care for the poor populations of Vellore town. It has been actively working among urban communities since 2002, with a focus on delivering Community Oriented Primary Care (COPC), for six poor urban communities since 2016.

**Relevant changes:**

During the first wave of COVID the LCECU team ensured patients with NCDs had uninterrupted primary care and medications by visiting them in their homes. The team also addressed food insecurity by organizing a daily lunch service for 600 people for over 2 months. In the second wave, the team responded to community needs by organizing and delivering home-based care to monitor patients affected by COVID-19.

**Lessons learned:**

The COVID-19 pandemic raises many questions about the preparedness of health systems for disasters that disproportionately affect marginalized populations globally. COVID-19 is only one of the many potential disasters, including non-communicable diseases, mental health problems, pollution, climate change, and lifestyle illness. There is an urgent need to study models of care that support vulnerable communities in an accessible, cost-effective, and patient-oriented way, particularly in low- and middle-income countries. This paper outlines lessons on how the LCECU team addressed disaster management:

1. The COVID-19 pandemic has highlighted the importance of primary care-based rapid response interventions in disaster management.

2. The LCECU model demonstrated the effectiveness of a primary care intervention based on pre-existing networks and familiarity between primary care teams and the community.

3. Establishing community-based health care *via* interdisciplinary teams, including community health workers, community volunteers, outreach nurses, and doctors, is key.

4. Addressing other social determinants of health, such as food insecurity, is an important component of care delivery.

## Background

With nearly 31 million confirmed cases and more than 410,000 deaths due to COVID as of July 13th, 2021, India was struck hard by two waves of the COVID-19 pandemic (1). The Indian government responded to the first wave with a nationwide lockdown on March 25th, 2020, controlling the infection rate (2). The second wave started in the middle of March 2021. Tamil Nadu, in southern India, was one of the worst affected in the country, with nearly 250,000 cases and over 33,000 deaths as of July 14th, 2021 (3). The surge in patients in the second wave resulted in a complete state lockdown from May 4th, 2021 to July 19th, 2021. The city of Vellore, in Tamil Nadu, has a population of 186,000. Vellore had over 16,000 cases in the first wave. During the second wave, the Corporation area reported 300 new cases per day in May 2021. To control the spread, the Vellore City Municipal Corporation, the civic body that governs the city, converted 85 streets to “containment streets”, blocking entry and exit of residents within barricades except for emergencies (4).

## Problem

The pandemic brought forward two main challenges for primary care for the urban poor that necessitated urgent response and adaptation of services. First, was the need to provide ongoing continuity of care to patients with Non-Communicable Diseases (NCDs). Diabetes, hypertension, dyslipidemia, physical inactivity, and obesity are higher in the urban area of Vellore than in rural areas (5). Second was the management of those infected with COVID-19, particularly during the second wave, when it was necessary, to shift care to homes given the lack of hospital beds regionally and nationally.

## Setting and intervention

The Christian Medical College, Vellore (CMC), a 3,000-bed institution spread over seven campuses with its motto “not to be ministered unto, but to minister”, started the Low-Cost Effective Care Unit (LCECU) in 1982 to respond to the need of the urban poor. The LCECU is a 48-bed unit under the Department of Family Medicine. It provides highly subsidized, accessible, and affordable primary and secondary health care to the poor communities of Vellore. The LCECU built a network of Community Health Volunteers—originally trained through a Community Based Rehabilitation Project in 2002 (6)—and further developed with the LCECU in 2016 (to focus on Community Oriented Primary Care in six poor communities in Vellore targeting a population of 10,000 residents. The interdisciplinary team included community health workers (CHWs), an outreach nurse and social workers who collectively engaged with the community, including training community volunteers, and conducting home visits and weekly community clinics alongside family and community medicine physicians from the LCECU. Secondary-level care was provided in the base hospital of LCECU, and those who need tertiary care are referred to the CMC hospital for subsidized care.

## Results

### Response to the first wave

During the first wave, the focus was on ensuring that patients with NCDs received their routine medications. Through the work of the LCECU over the years, a registry of patients with chronic diseases in these communities was developed. The CHWs knew where patients lived, what medications they were on, and when their prescriptions needed refilling. Initially, CHWs went to patient homes, checked their patient-retained health record of their medications and organized prescriptions for the patients. The patients could then come and pick up the medications from the LCECU. However, when some areas were contained, and entry and exits were blocked, the community volunteers maintained communication with the CHWs over mobile phone and the prescriptions were given to volunteers who gave them to the patients. Networking with the volunteers also allowed the LCECU to remain aware of health issues in the community and rapidly respond to them. The volunteers alerted the unit to loss of jobs and lack of income in the community. People were struggling to access food, and this became an unprecedented need for the unit to respond to. Local individuals and groups supported the unit in providing lunch for about 600 people a day for more than 2 months. Had our resources been greater we may have been able to provide more meals or address other social factors that affected quality of life of our patients. The decades spent building relationships and developing community networks allowed our team to respond to the acute crisis caused by COVID lockdowns and restricted mobility.

### Response to the second wave

As the second wave ravaged parts of India, various departments of the CMC coordinated and planned ways to mitigate the anticipated impact in Vellore. The program was called *UDHAVI* (meaning “help” in Tamil). It consisted of four areas: (1) triaging and providing telephone advice on COVID-19; (2) logistics that procured and distributed equipment (monitoring kits, oxygen concentrators, etc.); (3) counseling services for patients; and (4) home-based care for communities. When CMC learned that the local Indian Medical Association was planning similar telephone advice, we joined hands on the UDHAVI effort. This section describes the home-based care of the LCECU and its process.

#### Recruitment and training of community volunteers

LCECU held meetings in six service slum areas, explaining the need and plan for home-based care and recruiting community volunteers. The risk for volunteers was explained. Younger and vaccinated individuals were recruited. The LCECU provided COVID-related education and training on using the home-based care kits, which included pulse oximeters, thermometers, hand sanitizer and symptom monitoring sheets.

#### Identifying patients with COVID in the community

Patients were identified with the help of the volunteers by self-reporting and from the hospital lab database. Each patient who was eligible/opted for home-based care was met by the team, and one primary caregiver in the family was identified. The primary caregiver was taught to monitor the patient and complete the monitoring sheet. Caregivers contacted the CHW in their area twice daily with the monitoring data or sooner if red flags were noted. Families with smartphones and WhatsApp photographed the monitoring sheet and sent it to the CHW. However, the CHWs typically called the patients and their primary caregivers.

#### Home visits

The volunteers, CHWs, social workers and doctors visited patients daily. Follow-up was designed so that the volunteers would inform the doctors immediately if the patient needed escalated care. Otherwise, they reported a summary to the social worker who compiled the number of patients under care for discussion in the daily evening meeting. The team used WhatsApp for quick communication and coordination. The concept of confidentiality and shared confidentiality was explained and followed. The team documented findings and plans in the patient-retained charts, photographed it and then re-entered the same data on the electronic chart record by the next day at the base hospital. A few medical consultations with tertiary-level colleagues were held *via* phone. One cancer survivor with multiple co-morbidities who opted for home-based care, was given oxygen *via* concentrator, and visited daily in the home by the team. This included one visit by an internal medicine consultant and a respiratory technician. Though she passed away at home, the family was grateful for the palliative care provided and the opportunity to be with her during her last hours. This would not have been possible if she had been admitted to the hospital.

#### Review and planning meetings

The team met *via* Zoom daily to provide updates and discuss cases. Most of the patients cared for (34/37) required home-based care. One refused admission, one passed at home and one required admission for uncontrolled diabetes. Those needing medications such as inhaled budesonide, oral steroids, apixaban and supplemental oxygen were monitored by doctors directly as opposed to the CHWs. In addition to the care at home, the LCECU team secured funding through institutional fundraising efforts for patients needing admission at the main hospital for higher-level care.

The pandemic tested the network's resilience of community care programs the LCECU had built over the years and demonstrated the effectiveness of primary care based on community engagement and empowerment.

### Ongoing care

LCECU was identified as one of the outreach centers of CMC for providing free vaccines to the community. Initially the vaccines were provided in the unit and later in the six outreach communities with the active involvement of the volunteers and a health team from the LCECU. The outreach clinics were restarted once the second wave subsided.

## Discussion

One of the characteristics of a robust healthcare system is its ability to cope with sudden and unexpected health crises. The COVID-19 pandemic tested the tenacity of healthcare systems in every country in the world (7). Mortality was disproportionately higher in vulnerable populations, including the poor, elderly, migrants, minorities, and people living in densely populated areas. These groups have higher rates of NCDs, making them more vulnerable to COVID-19 infection and severe consequences of the disease and often fall through cracks in the healthcare systems (7–9). This article described how care of the urban poor in a city in southern India was adapted to meet the challenges of the pandemic. There were two major challenges that these communities faced during the pandemic; access to affordable, continuous care for those who had non-communicable diseases and a lack of hospital beds. Both required responses that deviated from the typical reactive hospital-based care model.

The lockdown and restricted movement of people could have had widespread implications for patients with chronic diseases had there not been an alternative arrangement to obtain regular care. The existing systems that LCECU had in place through its COPC program that blended public health with primary care acted as a safety net during this period. The network of volunteers, CHWs, outreach nurses, social workers and physicians caring for six defined vulnerable populations ensured that those who needed medical care received it, even during the strict lockdown. There were systems in place to identify those with chronic diseases requiring ongoing care and medications through home visits. Simultaneously, volunteers from the communities were constantly communicating with the CHWs in the team, alerting them to the urgent medical needs of community members. This went beyond the medical needs to address the social determinants of health. The LCECU played a key role in coordinating the supply meals to more than six hundred people for more than 2 months, when it became evident that many were struggling to eat due to loss of jobs and relatedly income.

The second wave of COVID infection overwhelmed the hospitals in Vellore. The poor from the town turned up at LCECU with fever, cough and shortness of breath. CMC had developed a system to help the communities around the institution. Using an interdisciplinary team-based approach, home care was provided for many patients. What enabled the LCECU team to quickly respond and deliver this unconventional method of care was their longstanding relationship with the communities. This was primarily due to the work of the CHWs and their engagement of volunteers from the communities. The LCECU had worked intensively in the six poor areas through their COPC program and built deep trust relationships over many years. The benefits of networks in care delivery, where the community is involved through its volunteers who are trained regularly to address the various needs, have been highlighted in several articles (10–12). Similarly, they highlight the importance of investing in relationships of trust with the community.

In general, there has been little published on existing models of care for the management of NCDs during COVID-19 or other disasters. However, the integration of CHW's as key players in emergency responses is being advocated globally (13). Similar to the LCECU example, the community health clinic program in Bangladesh found that CHW's made significant contributions ensuring essential health services continued during the COVID-19 pandemic (14).

The steps taken by LCECU in actively reaching out to the six areas with visits by various members of the outreach teams and starting outreach clinics, prepared the ground for rapid and effective responses when the pandemic hit them. This reiterates the need for responses to disasters that are based in primary care and emphasizes the important role of interdisciplinary teams and community engagement.

## Conclusions

The COVID-19 pandemic raises many questions about the preparedness of health systems for disasters that affect all sections of people globally. COVID-19 is one of the many disasters affecting people worldwide, including non-communicable diseases, mental health problems, pollution, and climate change. There is an urgent need to study models of care that can address these in an accessible and cost-effective manner, especially in low- and middle-income countries. This paper highlights: (1) the critical role primary care plays in responding to disasters; (2) the effectiveness of interdisciplinary team-based primary care; (3) the benefits of a COPC model of care based on community engagement and building networks and familiarity between primary care staff and community volunteers; and (4) the important role of addressing the social determinants of health.

## Data availability statement

The raw data supporting the conclusions of this article will be made available by the authors, without undue reservation.

## Author contributions

SA led the strategic planning exercise for restricting the outreach and community services of LCECU through Community Oriented Primary Care, gave leadership to the follow up of non-communicable diseases during the pandemic, and contributed to the manuscript. SJ led the care of patients during the second wave including putting systems in place of home based care and contributed to the preparation of the manuscript. AG involved in discussions about the manuscript, made corrections, and changes to the drafts. SB contributed significantly to preparation of the manuscript and finalize the words used to describe the response to the disaster. MK made significant contribution to writing the manuscript. PM and AF involved in the ground work of the response including home visits and follow up of patients with COVID-19. All authors contributed to the article and approved the submitted version.
